# Rapid development of a spinal epidural hematoma following thoracic epidural catheter removal in an esophageal carcinoma surgical patient: a case report

**DOI:** 10.1186/s40981-016-0060-7

**Published:** 2016-11-14

**Authors:** Takeshi Umegaki, Kiichi Hirota, Sayaka Ohira, Takeo Uba, Munenori Kusunoki, Akihisa Okamoto, Kenichiro Nishi, Takahiko Kamibayashi

**Affiliations:** 1 Department of Anesthesiology, Kansai Medical University Hospital, Kansai Medical University, 2-3-1 Shin-machi, Hirakata, Osaka 573-1191 Japan; 2 Department of Human Stress Response Science, Institute of Biomedical Science, Kansai Medical University, Osaka, Japan

**Keywords:** Spinal epidural hematoma, Thoracic epidural catheter, Esophageal carcinoma

## Abstract

**Background:**

The occurrence of spinal epidural hematomas associated with the use of epidural catheters is relatively rare. Furthermore, it is unusual for hematoma-associated neurological symptoms to occur within 15 min of removing a catheter. Here, we report our experience with an esophageal carcinoma surgical patient who developed an epidural hematoma almost immediately after catheter removal, resulting in paralysis of his lower extremities. The patient achieved full neurological recovery following prompt diagnosis and surgical intervention.

**Case presentation:**

A 68-year-old man was admitted with esophageal carcinoma and underwent video-assisted thoracoscopic esophagectomy followed by posterior mediastinal gastric tube reconstruction. During surgery, the patient was administered both general and epidural anesthesia. The epidural catheter was inserted approximately 5 cm into the epidural space at the Th6–7 level. The patient was extubated the following day in the general intensive care unit. Two days after surgery, the d-dimer level was high at 36.9 μg/mL (reference range 0–0.9 μg/mL), and we decided to administer an anticoagulant (enoxaparin sodium) to prevent thrombosis. The epidural catheter was removed 2 h prior to the scheduled administration of enoxaparin sodium. However, the patient reported a complete lack of strength in his lower extremities 15 min after catheter removal. Upon examination, the manual muscle testing score was 1 out of 5, and the patient experienced impaired touch sensation and cold sensation below Th4. An emergency magnetic resonance imaging scan was performed 2 h after catheter removal, which revealed a possible spinal epidural hematoma spreading from Th3 to Th6. Three hours after catheter removal, we began emergency surgery to evacuate the hematoma, which had spread to Th7. After surgery, the patient showed improvements in touch sensation, cold sensation, and motor function. The patient was able to walk 2 days after hematoma removal.

**Conclusions:**

It is highly unusual for a spinal epidural hematoma to develop so rapidly after the removal of an epidural catheter. This case emphasizes the need for vigilant patient monitoring, rapid diagnosis, and prompt surgery to ensure adequate neurological recovery in these patients.

## Background

Several studies have described the occurrence of spinal epidural hematomas associated with the placement and removal of epidural catheters [1–4]. Due to the association between the duration of hematoma-induced spinal cord compression and the degree of neurological symptoms [5], prompt diagnosis and surgical treatment are critically important determinants of neurological recovery. As the progression of severe symptoms generally occurs after an epidural catheter has been placed for more than 6 h [6], it is exceedingly unusual for hematoma-induced paralysis to occur within 15 min of catheter removal. Here, we report our experience of an esophageal carcinoma surgical patient who developed paralysis of his lower extremities due to an epidural hematoma that had occurred within 15 min after removing a thoracic epidural catheter. Neurological recovery of the patient was accomplished through prompt diagnosis and surgical treatment.

## Case presentation

The patient was a 68-year-old man of 166 cm height and 60 kg weight who was diagnosed with an esophageal carcinoma (T3N1M0, stage III). We performed video-assisted thoracoscopic esophagectomy followed by posterior mediastinal gastric tube reconstruction. There was no indication of coagulopathy prior to surgery. The activated partial thromboplastin time (APTT) was 27.2 s (reference range 23–35 s), prothrombin time (PT) was 108.0% (reference range 75–130%), and platelet count was 24.4 × 10^4^/μL (reference range 14–34 × 10^4^/μL).

Anesthesia was provided using a combination of general and epidural anesthesia. For the latter, the epidural catheter was inserted at the Th6–7 level and advanced approximately 5 cm into the epidural space. We did not observe any neurological symptoms, hemorrhage, or dural puncture during catheter implantation. Propofol and fentanyl were administered intravenously to induce anesthesia, and endotracheal intubation was carried out after rocuronium administration. Anesthesia was maintained using oxygen/air/desflurane with additional rocuronium as needed. For intraoperative analgesia, a total of 20 mL of 1.5% lidocaine was administered via the epidural catheter. Following video-assisted thoracoscopic resection of the esophagus, we reconstructed the gastric tube through laparotomy and performed cervical esophageal anastomosis using the posterior mediastinal route. In addition, we also performed regional lymphadenectomy (in three nodal regions) and enterostomy. The operation time and anesthesia time were 405 and 536 min, respectively. Blood loss during surgery was 221 mL.

After surgery, the patient was moved to the general intensive care unit and was extubated the following day; no neurological deficits were observed at this point. Two days after surgery, APTT, PT, and platelet count were within normal limits. However, high levels of d-dimer (36.9 μg/mL; reference range 0–0.9 μg/mL) indicated a need to prevent thrombosis, and we decided to administer enoxaparin sodium (Clexane™, SANOFI, Tokyo, Japan), a low-molecular-weight heparin, as an anticoagulant. At that time, fibrinogen level was 419 mg/dL (reference range 150–350 mg/dL), APTT was 29.1 s, PT was 81.4%, and platelet count was 5.4 × 10^4^/μL. The epidural catheter was removed 2 h before the scheduled administration of enoxaparin sodium. The patient was placed in the decubitus position for catheter removal, and the catheter was removed smoothly without resistance.

**Fig. 1:**
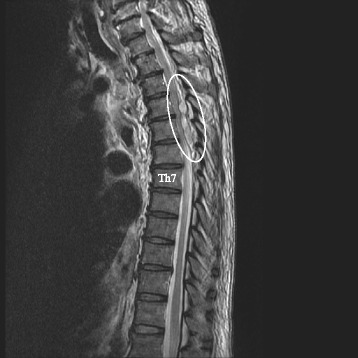
T2-weighted sagittal magnetic resonance image. The spinal epidural hematoma (*circle*) is indicated by the hyperintense region in the epidural space from Th3 to Th6

**Fig. 2:**
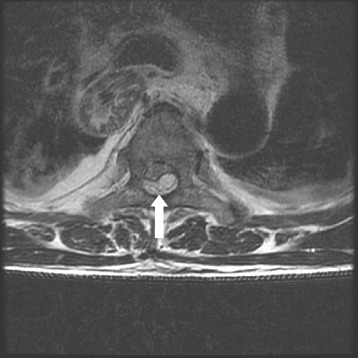
T2-weighted axial magnetic resonance image at the Th5 level. The spinal epidural hematoma (*arrow*) is observed to be pressing the spinal cord toward the ventral side

Fifteen minutes after catheter removal, the patient complained of a complete lack of strength in both of his lower extremities. The patient had a manual muscle testing score of 1 (out of a possible 5), and he reported a lack of touch sensation and cold sensation below Th4. Due to this unexpected development, an emergency magnetic resonance imaging (MRI) scan was performed 2 h after catheter removal, which indicated a possible spinal epidural hematoma spreading from Th3 to Th6 (Fig. 1). As shown in Fig. 2, the hematoma was pressing the spinal cord toward the ventral side. The patient experienced involuntary movements in his lower extremities during MRI scanning. Three hours after catheter removal, we began emergency surgery to remove the hematoma. Within an hour, we had confirmed and removed a hematoma that spanned from Th3 to Th7; the hematoma had spread to another spinal segment since the MRI scan had been performed. After surgery, the patient demonstrated gradual improvements in touch sensation, cold sensation, and motor function. The patient was able to walk 2 days after hematoma removal, and involuntary movements ceased after 7 days.

### Discussion

Catheter-associated spinal epidural hematomas can occur during catheter placement or removal, and incidence has been reported to range from 2 to 20 cases per 100,000 epidural procedures [4, 7, 8]. Despite their relatively low incidence, these hematomas are widely recognized by anesthesiologists as a possible deleterious complication of epidural anesthesia. Furthermore, the development and progression of hematomas associated with epidural anesthesia is relatively slow and is generally characterized by symptoms such as dorsalgia, impaired motor function (paraplegia), and sensory abnormalities; intense pain also occurs in many cases [6]. In the present case, the patient rapidly developed paraplegia and sensory abnormalities without any intense back pain. Rapid diagnosis was facilitated through the use of T2-weighted MRI, which produced clear visualization of the hematoma. Previous investigations of the time course of spinal epidural hematoma development reported that paraplegia can occur as early as 1 h after catheter removal, with some cases developing symptoms after more than 24 h [1, 9]. In contrast, the case described here is unusual in the rapid manifestation of neurological symptoms after catheter removal.

The epidural cavity, which lies between the lamina externa and lamina interna, contains loose connective tissue, adipose tissue, and venous plexuses. Therefore, the veins in the epidural cavity constitute the primary source of epidural hematomas. In the report by Gulur et al., epidural hematomas occurred in patients with abnormal coagulation parameters after the epidural catheter had been removed [2], indicating the importance of conducting follow-up examinations. Although our case patient did not develop paraplegia or sensory abnormalities during the 2 days when the epidural catheter was implanted, these symptoms rapidly developed after catheter removal, suggesting that a venous plexus was damaged during the removal process. This supposition was supported by the absence of arterial hemorrhage or arteriovenous malformation during surgery. However, it is interesting that the degree of hemorrhage from a vein could result in such rapid onset of neurological symptoms.

The following abnormal coagulation parameters during the perioperative period have been identified as risk factors for epidural hematoma occurrence: APTT >35 s, international normalized ratio of PT >1.5, and platelet count <10 × 10^4^/μL [2]. However, our case patient fulfilled none of these criteria for abnormal parameters. The levels of d-dimers, a fibrin degradation product, generally increase in patients with thrombotic disease or after undergoing surgery [10]. Despite the usefulness of d-dimer levels in the exclusion of deep-vein thrombosis [11], there is also a need to perform ultrasonography of the veins in the lower extremities for accurate diagnoses. Although our case patient underwent ultrasound scanning, the field of view did not indicate any sign of thrombosis.

Studies have shown that patients with epidural hematomas should undergo surgical intervention within 8 h from the onset of symptoms to allow for neurological recovery [5]. In our case patient, we had detected and surgically evacuated the hematoma within 4 h after catheter removal. It is likely that the rapid response and surgical intervention were crucial in facilitating neurological recovery.

It should be noted that the increasingly prevalent use of postoperative anticoagulation therapy in recent years has provided the option of utilizing nerve blocks in place of epidural anesthesia. Peng and Wang reported that the use of a thoracic paravertebral intercostal nerve block was able to achieve satisfactory postoperative analgesia in esophageal cancer patients [12]. In our institution, however, analgesia control for thoracoabdominal surgery is generally performed using epidural anesthesia. The guidelines of the American Society of Regional Anesthesia and Pain Medicine for anticoagulant therapy recommend the use of a low-molecular-weight heparin (such as enoxaparin sodium) at least 4 h after epidural catheter removal [13]. In accordance with this practice, we had planned to remove the epidural catheter before administering the anticoagulant.

## Conclusions

Here, we report our experience of an exceedingly unusual case that rapidly developed a spinal epidural hematoma following the removal of an epidural catheter, which resulted in paralysis below Th4. The implications of this case emphasize the need for vigilant patient monitoring, rapid diagnosis, and prompt surgery to ensure adequate neurological recovery.

## Abbreviations

APTT: Activated partial thromboplastin time
MRI: Magnetic resonance imaging
PT: Prothrombin time
